# Moralised Eating and Identity Work in Digital Wellness Culture: A Critical Discourse Analysis of Orthorexia-Related Narratives

**DOI:** 10.1007/s11013-026-10011-6

**Published:** 2026-07-27

**Authors:** Omer Horovitz

**Affiliations:** Tel Hai Academic College, Kiryat Shmona, Israel

**Keywords:** Orthorexia nervosa, Moralisation, Discursive identity fusion, Self-concept rigidity, Clean eating, Social media

## Abstract

Orthorexia nervosa (ON) is increasingly understood as a morally saturated health orientation in which dietary restraint is organised through ideals of purity, discipline, and self-worth. Building on social theory that treats health as a cultural project and a site of identity governance, this study theorises ON as moral identity work enacted and contested in digital environments. Using a large-scale qualitative design integrating thematic corpus mapping and Critical Discourse Analysis (CDA), 1,100 texts from Reddit, YouTube, and blogs (2020–2025) were analysed; an intensified CDA subset (n=80) traced how key meanings were rhetorically accomplished. Across platforms, moralised purity/contamination vocabularies and identity-relevant self-positioning were common but genre-sensitive. In the CDA subset, a recurring configuration framed restrictive eating as (a) morally evaluative, (b) identity-defining and non-negotiable, and (c) defended via boundary work and resistance to pathologisation; recovery narratives more often disrupted these binaries and decoupled selfhood from dietary purity. Extending existing accounts that document morality narratives around ON, the analysis specifies the discursive mechanisms and genre- and platform-sensitive configurations through which moralisation becomes identity-defensive and resistant to critique. These findings clarify the discursive mechanisms through which ON becomes intelligible and defensible within digital wellness culture, with implications for prevention and care focused on moralised self-concept and digitally reinforced rigidity.

## Introduction

Orthorexia Nervosa (ON) has increasingly been conceptualised as a culturally sanctioned yet psychologically consequential form of restrictive eating, characterised by an obsessive commitment to dietary purity, health, and moralised food practices (Horovitz & Argyrides, 2023). ON extends beyond nutritional concern to encompass rigid rules, moral absolutism, and identity-defining beliefs about food (Brytek-Matera, 2021; Hanganu-Bresch, 2020). Although ON remains absent from formal diagnostic systems such as the DSM-5-TR (Diagnostic and Statistical Manual of Mental Disorder, 2022), growing empirical and theoretical literature has led some scholars to propose ON as a potentially distinct eating-related presentation characterised by health-focused restriction, dietary rigidity, and moralised beliefs about food, although this position remains contested (Horovitz & Argyrides, 2023; Koven & Abry, 2015).

However, the nosological status of ON remains actively debated. While some researchers argue that orthorexia is distinguishable from other eating-related conditions because of its emphasis on health, purity, and dietary correctness rather than weight or appearance concerns, others have questioned whether ON represents a truly distinct disorder or whether it substantially overlaps with existing diagnostic constructs, particularly AN, obsessive–compulsive disorder (OCD), obsessive–compulsive personality traits, and perfectionism (Cena et al., 2019; Dunn & Bratman, 2016; Koven & Abry, 2015). Clinical and qualitative studies suggest that some individuals who identify with orthorexic concerns also report body dissatisfaction, restrictive eating practices, fear of weight gain, and self-evaluative processes commonly associated with anorexia nervosa (AN), leading some scholars to question whether at least a subset of orthorexia presentations may represent variant expressions of restrictive eating pathology rather than a wholly separate condition (Cena et al., 2019; Koven & Abry, 2015). Consequently, scholars increasingly view ON as a heterogeneous phenomenon with potentially overlapping pathways rather than a uniformly distinct clinical entity.

In addition, some scholars have noted that orthorexia-related presentations may extend beyond dietary restriction alone and become embedded within broader healthism-oriented lifestyles involving intensive exercise, fitness optimisation, self-monitoring, and bodily discipline (Crawford, 1980; Hanganu-Bresch, 2020; Lupton, 2012, 2013). In such accounts, food regulation forms part of a wider project of health management and self-improvement rather than an isolated concern with eating behaviour (Crawford, 1980; Lupton, 2013; Musolino et al., 2015). These observations further support the view that orthorexia is a heterogeneous phenomenon that may be expressed differently across cultural and social contexts (Cena et al., 2019; Koven & Abry, 2015; Musolino et al., 2015).

The present study does not seek to resolve these diagnostic debates. Instead, it examines how orthorexia-related meanings, identities, and moral evaluations are constructed and negotiated within digital environments, regardless of their ultimate nosological classification. Importantly, the present study adopts a discursive rather than diagnostic orientation. Whether orthorexia ultimately emerges as a distinct clinical entity, a transdiagnostic syndrome, or a culturally specific manifestation of existing eating-disorder and obsessive-compulsive processes is beyond the scope of the analysis. Instead, the focus is on how orthorexia-related meanings are produced, circulated, defended, and contested within digital environments. This approach allows examination of the social and moral organisation of orthorexia-related discourse without presupposing any particular diagnostic resolution.

Digital wellness culture provides an important context for understanding the contemporary visibility of orthorexia-related discourse. Building on broader ideologies of healthism, digital wellness culture refers to online environments in which health is framed as an ongoing project of self-optimization achieved through dietary management, self-monitoring, lifestyle discipline, and continuous self-improvement (Crawford, 1980; Lupton, 2015). Within these spaces, practices such as clean eating, food tracking, detoxification, supplementation, and biohacking are frequently presented not merely as health behaviours but as indicators of responsibility, self-control, and moral commitment. Social media platforms further amplify these ideals by rewarding visibility, consistency, and personal testimony, creating environments in which health-related identities can be publicly performed, validated, and reinforced (Lupton, 2015). Scholars have argued that such environments encourage the moralisation of health and the responsibilisation of individuals for bodily outcomes, making digital wellness culture particularly relevant for understanding how restrictive eating practices become tied to virtue, identity, and self-worth (Crawford, 1980). Orthorexia-related discourse frequently circulates within these contexts, where distinctions between healthy eating, self-improvement, and pathology are actively negotiated.

This article advances orthorexia discourse as an exemplary case through which broader processes of health moralisation, responsibilisation, and identity governance can be examined. Rather than treating orthorexia as merely one manifestation of healthism among others, the analysis shows how orthorexia-related discourse can be organised into a particularly rigid and self-stabilising moral–identity configuration (Lupton, 2012, 2013). Orthorexia is therefore approached not only as an expression of contemporary healthism but as a distinctive discursive formation in which dietary practice becomes tightly bound to moral worth, self-definition, and resistance to critique. Where existing scholarship has compellingly documented morality narratives and psycho-politics surrounding ON (e.g. ‘clean’ food as virtue; moral regulation of the self), this study adds analytic specificity by identifying the discursive mechanisms and recurrent configurations through which moralisation is accomplished, defended, and occasionally disrupted in platformed settings (Fixsen et al., 2020; Hanganu-Bresch, 2020; Horovitz, 2025).

A recurring theme across qualitative accounts is the ego-syntonic character of orthorexic cognition, in which restrictive practices are experienced as virtuous, rational, and ethically justified rather than problematic (Cascino et al., 2019; Roncero et al., 2013). In contrast to eating-disorder presentations primarily organised around body dissatisfaction or appearance concerns, orthorexia-related narratives often foreground integrity, discipline, health knowledge, and moral responsibility (Cheshire et al., 2020; Nevin & Vartanian, 2017). These features contribute to diagnostic ambiguity and may help explain why orthorexic practices are frequently maintained despite distress or impairment.

From this perspective, orthorexia can be approached analytically as a form of moral–identity work, in which dietary practices function as symbolic markers of selfhood, virtue, and epistemic certainty. Food choices become more than behaviours to be regulated; they operate as moral statements about who one is, what one values, and where one stands in relation to others (Aquino & Reed, 2002; Sneijder & Molder, 2009). This orientation does not presuppose that all individuals who engage in restrictive eating hold a fused or rigid identity structure, but it highlights a culturally available configuration through which such practices can become identity-defining and resistant to critique.

The present study does not seek to adjudicate the clinical status of ON or to advance a formal psychological model. Instead, it examines how specific discursive resources, moral evaluation, self-positioning, boundary work, and resistance to pathologisation render orthorexia intelligible, legitimate, and defensible within digital wellness cultures. By focusing on publicly available online narratives, the analysis treats orthorexia-related discourse as situated social performance shaped by platform norms, audience expectations, and broader cultural ideologies of health and purity. Accordingly, this study asks:

1. What recurrent moral-evaluative vocabularies and narrative forms appear in orthorexia/clean-eating self-labelling texts across platforms (corpus mapping)?
2. In a smaller, information-rich subset, what discursive mechanisms (e.g. moral authorisation, boundary work, resistance to pathologisation) make restrictive eating appear identity-defining and justified (CDA)?
3. Where and how do texts explicitly contest these moral binaries or decouple identity from dietary purity (counter-patterns)?

To reduce theoretical overextension, the analysis is anchored primarily in CDA and moral/identity positioning (Aquino & Reed, 2002; Fairclough, 2013), using “identity fusion” as an interpretive label only when specified discursive criteria are met (see Methods). Here, “identity fusion” is used for analytic economy as a discursive shorthand (identity linkage + non-negotiability + boundary/defense), not as an endorsement of psychological identity-fusion models or a claim about underlying mental states. Alternative labels (e.g. “identity anchoring,” “moral self-binding”) were considered; “identity fusion” is retained because it most directly captures the observed linkage between self-definition and defended non-negotiability in the texts.

## Methods

### Design and Methodological Approach

This study employed a large-scale qualitative research design integrating Critical Discourse Analysis (CDA) and thematic analysis to examine the digital construction of ON as a morally infused and ideologically anchored identity.

Two-tier analytic design. (1) Corpus mapping via thematic analysis across all texts identified recurring topics, evaluative patterns, and platform/genre differences. (2) Intensified CDA on an information-rich subset examined how those meanings were rhetorically accomplished (e.g. moral evaluation, identity positioning, legitimation, boundary work). This structure is used to prevent “whole-corpus” generalisation from subset-level discursive mechanisms.

Operational criteria for “identity-fusion positioning” (discursive, not psychological). In the intensified CDA subset, a text was coded as showing identity-fusion positioning only when it contained all three of the following features:

1. *Identity linkage *Explicit self-definition tied to diet (e.g. “I am / I’m not the kind of person who…”).
2. *Non-negotiability* Modal absolutism or inevitability (e.g. “must,” “always/never,” “can’t be me otherwise”).
3. *Defense/boundary work *Justificatory contrast with an “other” (e.g. “people who eat normally don’t get it”) or explicit resistance to critique/pathologisation.

Epistemological orientation. The study is situated within a social constructionist epistemology, with sensitivity to the material consequences of discourse. Claims are limited to discursive construction and cultural intelligibility. Reflexive memos were maintained throughout the analysis to surface assumptions and to identify counter-examples. To mitigate the risk of confirmatory interpretation, competing readings (e.g. moral identity work, anxiety regulation, platform performance incentives) were considered in parallel. Interpretations reported in the Results specify when the moral–identity reading provided the strongest explanatory fit and when alternative readings better accounted for a given discursive feature. Although the corpus is large, its size is used to support discursive breadth and thematic saturation rather than representativeness, prevalence estimation, or population-level inference.

Although the study was informed by prior theoretical interests in moralisation, identity positioning, and healthism, thematic patterns and discursive configurations were developed iteratively through engagement with the data rather than being imposed a priori.

### Data sources and Sampling

The analytic corpus consisted of approximately 1,100 publicly accessible digital texts drawn from Reddit, YouTube, and blogs/personal essays. Sampling followed a purposive strategy designed to maximise discursive richness, narrative depth, and conceptual relevance rather than representativeness. Reddit was selected because its pseudonymous structure facilitates extensive self-disclosure, peer support, and discussion of health-related struggles. YouTube was included because it hosts highly visible wellness, recovery, and lifestyle narratives that combine personal testimony with audience-oriented health communication. Blogs and personal essays were selected because they provide longer-form reflective accounts that often permit greater narrative elaboration than social media posts.

Texts were identified using platform-native searches and supplementary Google searches combining terms including orthorexia, orthorexia nervosa, clean eating, food purity, healthy eating obsession, food anxiety, and orthorexia recovery. Blogs and personal essays were screened manually for relevance and narrative depth. Inclusion criteria were: (a) explicit discussion of orthorexia, clean eating, food purity, restrictive health-oriented eating, or recovery from such practices; (b) sufficient narrative content to support qualitative analysis; (c) public accessibility without registration requirements; and (d) English-language content. Exclusion criteria included advertisements, commercial nutrition marketing, recipe-only pages, duplicate texts, inaccessible content, and purely informational health materials lacking personal, evaluative, or identity-related discourse.

**Fig. 1:**
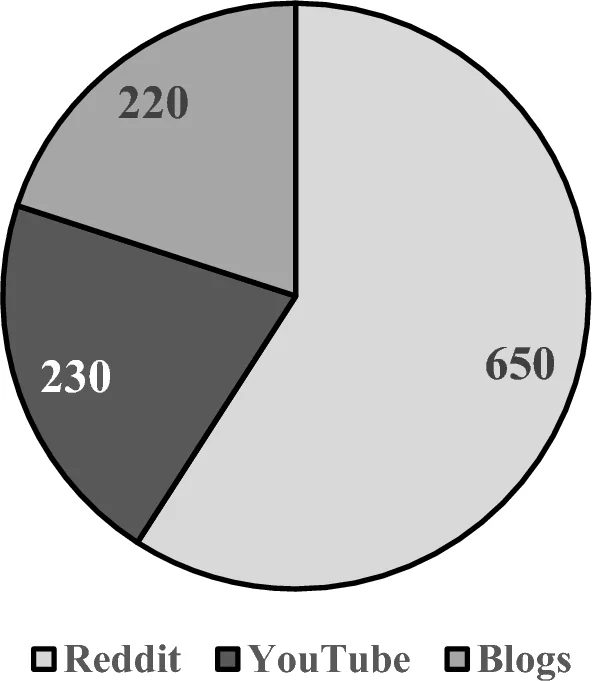
Composition of the digital corpus analysed across platforms

Subset selection for intensified CDA. The intensified CDA subset comprised approximately 80 texts (Reddit: 40; YouTube: 20; blogs/essays: 20). Selection was stratified by (i) platform, (ii) narrative orientation (recovery vs lifestyle advocacy/education vs ambivalent reflection), and (iii) density of moral-evaluative language and explicit self-positioning. The subset intentionally included counter-cases that minimised moral language or explicitly rejected “good/bad” framing (see Fig. 1).

Figure 1 displays the distribution of the approximately 1,100 publicly available digital texts included in the analytic corpus, organised by platform (Reddit, YouTube, and blogs/personal essays). The figure illustrates the relative contribution of each platform to the overall dataset and provides methodological context for the discursive analyses reported in the Results section. The corpus spans the period from 2020 to 2025 and was assembled using purposive sampling to maximise discursive richness rather than statistical representativeness. Figure 1 is presented for descriptive orientation to the dataset, not as an inferential or causal claim.

The CDA component was informed by critical discourse analytic approaches that examine the relationship between textual features, discursive practices, and broader sociocultural processes 22. Analysis moved iteratively between close examination of linguistic and rhetorical features within individual texts and consideration of the wider cultural contexts in which those meanings were produced and circulated. Particular attention was given to moral evaluation, identity positioning, legitimation strategies, boundary construction, and resistance to pathologisation. This approach enabled examination not only of what participants said about food and health, but also of how these meanings were organised within broader discourses of healthism, self-discipline, moral responsibility, and digital wellness culture.

### Data Collection Procedures

Data were collected between January 2025 and October 2025 using platform-native search functions to ensure compliance with platform terms of service. Reddit posts were manually archived by copying post titles and bodies into a secure dataset; usernames, profile links, and other identifying information were excluded at the point of collection. YouTube videos were identified through keyword searches, and transcripts were obtained using publicly available transcript-generation tools or, where necessary, manual transcription. Blog texts were archived by copying the main body of each article, excluding comments and unrelated content. Each text was logged with metadata including platform, publication date, and general context (e.g. recovery-oriented, lifestyle advocacy, reflective narrative). No interaction with content creators occurred at any stage of the research. All materials were treated as “public texts” while recognising that contributors may not anticipate academic analysis; this consideration informed anonymisation and paraphrasing decisions (see Ethics).

### Data Analysis

Thematic analysis (Braun & Clarke, 2006) was conducted across the full corpus to identify recurring patterns related to moralised language, identity claims, rigidity, and emotional framing. CDA (Dijk, 1993; Fairclough, 2013) was applied to the intensified subset.

*Analytic demonstration and evidentiary strategy.* Because verbatim quoting can facilitate re-identification, illustrative examples are presented as short “composite excerpts” that preserve discursive structure (moral evaluation → self-positioning → defense) without being searchable. These composites are used only to demonstrate discursive mechanisms, not to attribute stable motives or diagnoses to any individual.

### Ethical Considerations

The study relied exclusively on publicly available online content, and no interaction with users or content creators took place. In accordance with established ethical guidelines for digital research, the following safeguards were implemented:

- Only content accessible without passwords, memberships, or private group access was included.
- Usernames and identifying details were omitted, paraphrased, or removed entirely;
- Quoted material was minimised and handled in a manner consistent with fair use and the protection of potentially vulnerable individuals.

Given the non-interactive use of public data, the study was deemed exempt from formal institutional ethics review, consistent with established guidance for internet-mediated research see: (Ethics Guidelines for Internet-Mediated Research, 2021; Eysenbach & Till, 2001). Because eating-disorder-related discourse may involve vulnerability and unintended exposure, the analysis was guided by interpretive responsibility: claims were framed at the level of discursive patterns rather than attributing stable motives or diagnoses to individuals. Where illustrative examples were needed, the study used (a) short, non-searchable paraphrases and (b) removal of platform-specific or personally identifying markers to reduce re-identification risk. Paraphrasing aimed to preserve the analytic features under discussion (e.g. moral binaries, “I am” identity claims) while preventing traceability through verbatim searching.

The analysis also recognised the ethical asymmetry between public availability and perceived privacy: even when content is technically public, contributors, particularly those describing distress or recovery, may not anticipate scholarly analysis or secondary circulation. This concern informed a “minimum necessary exposure” approach: usernames and identifying details were removed at collection; distinctive phrasing and biographical specifics were softened; and illustrative material was presented as short, non-searchable paraphrases or composites that preserve discursively relevant features (moral evaluation, identity positioning, boundary work) while reducing traceability.

## Results

The final analytic corpus comprised approximately 1,100 publicly available digital texts, distributed across three platforms: Reddit (n = 650), YouTube (n = 230), and blogs or personal essays (n = 220). Texts spanned the period from 2020 to 2025.

Across platforms, moral-evaluative vocabularies (purity/contamination; discipline/failure) and self-monitoring narratives were common, but their functions varied by genre (e.g. recovery reflection vs “what I eat” performance vs advice/education). Platform affordances also shaped these performances: Reddit posts were often dialogic and advice-seeking, YouTube narratives more overtly audience-facing and demonstrative, and blogs more elaborated and reflective. Counter-patterns, such as the explicit rejection of moral binaries and the decoupling of identity and diet, were especially evident in recovery-oriented texts. Intensified CDA (subset) identified a recurring configuration, not universal, but analytically coherent, through which restrictive eating is rendered morally legitimate and identity-defining. Four interrelated discursive domains are reported below, with mechanisms specified. As detailed in Domains 2–4, the configuration is ‘self-stabilising’ in the discursive sense that evaluative binaries are coupled to identity claims and then defended via boundary work and resistance to pathologisation, making counter-readings (flexibility, ambivalence, clinical problematisation) interactionally harder to sustain.

### Thematic and Discursive Findings

The analysis identifies a recurrent and culturally salient discursive configuration through which orthorexia-related practices are framed as morally meaningful and identity-relevant, rather than as a narrowly behavioural eating disturbance. Four interrelated discursive domains emerged.

### Moralisation of Food and Dietary Purity

Across the corpus, food was frequently framed using moral binaries. Foods were routinely classified as “clean,” “pure,” or “safe” in contrast to “toxic,” “dirty,” or “harmful,” with deviations framed as lapses requiring correction.

Illustrative examples included narratives describing refined sugar as “poison,” processed foods as “toxic” or “contaminating,” and dietary deviations as requiring “resetting,” “cleansing,” or renewed discipline. In lifestyle-oriented texts, foods perceived as natural, organic, or minimally processed were frequently associated with responsibility, purity, and self-control, whereas processed foods were linked to loss of control, bodily harm, or moral compromise.

In the intensified subset, purity terms were commonly linked to self-audit sequences (evaluation of intake → moral affect such as guilt/pride → corrective action). This mechanism appeared more consistently in advocacy/performance genres than in recovery reflection. A meaningful minority explicitly challenged “good/bad” food labels, reframing purity binaries as harmful or stigmatising.

### Identity Positioning and Non-negotiability

A central pattern was explicit identity linkage (e.g. “I am someone who eats clean”). Totalising language (“always,” “never,” “must”) appeared frequently. In subset texts that met the operational criteria, dietary rules were constructed as necessary for preserving a valued sense of self, making change narratable as identity loss rather than as behavioural flexibility. Recovery texts more often narrated identity decoupling (e.g. “this is something I do, not who I am”) and treated absolutist language as a symptom to be challenged.

Common formulations included statements resembling “this is who I am,” “healthy eating is part of my identity,” and “I could not be myself if I abandoned these practices.” Such constructions positioned dietary behaviour as a marker of personal integrity and self-definition rather than a flexible health-related choice.

### Boundary Work and Social Reinforcement

Digital platforms functioned as reinforcement environments. Users narrated routines and lapses in ways that invited affirmation and guidance. The identity-defining configuration often relied on contrastive positioning (disciplined self vs uninformed/undisciplined others), which served to legitimise rigidity and render it less vulnerable to critique. The same moves also aligned with platform incentives (audience retention, authenticity norms), suggesting that not all “virtue signaling” should be read as moral identity per se.

Several narratives contrasted disciplined eaters with “uninformed,” “careless,” or “mainstream” eaters, constructing symbolic boundaries between those perceived as committed to health and those viewed as lacking knowledge, discipline, or concern. These distinctions often functioned to legitimise dietary rigidity while reducing the credibility of criticism.

### Resistance to Pathologisation

Despite acknowledgement of distress, many narratives resisted pathologisation. Diagnostic labels were rejected or minimised, while behaviours were defended as rational and ethically justified. In subset texts meeting identity-fusion criteria, distress was frequently narrated as a cost of integrity, maintaining commitment despite perceived costs or impairment. Illustrative examples included narratives in which restrictive eating was defended through appeals to health, self-discipline, or nutritional expertise. Challenges to these practices were frequently reframed as misunderstandings of what constituted “real” health, allowing participants to maintain the legitimacy of dietary restriction despite acknowledging distress, inconvenience, or social conflict. Such formulations functioned to preserve the moral legitimacy of the practice while resisting its interpretation as pathological. In contrast, recovery narratives more often re-label “discipline” as impairment and use clinical language to destabilise moral legitimacy.

### Worked analytic demonstration

*Composite A (moral evaluation → identity linkage → defense):* “I try to keep things clean; processed food feels toxic. I’m not the kind of person who eats that way. People say it’s extreme, but they don’t understand what real health takes.”

*Composite B (confession → recommitment → audience validation):* “I slipped and ate something I shouldn’t. Tomorrow I’ll reset and be strict again. I need to stay on track; this matters to me.”

*Composite C (counter-pattern: decoupling + critique):* “Calling food ‘good’ or ‘bad’ kept me trapped. I’m learning to eat without moral grades. I can care about health without making it my identity.”

These composites illustrate the mechanisms coded in CDA, evaluation, self-positioning, boundary/defense, while preserving non-searchability

## Discussion

**Fig. 2:**
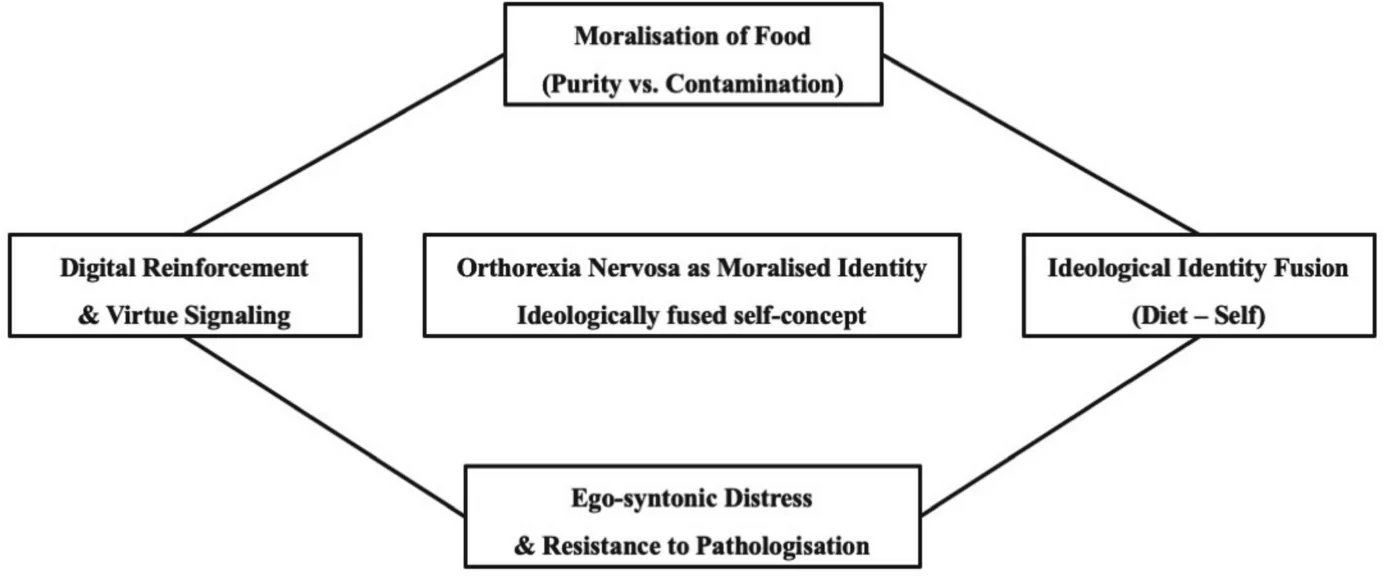
Integrative discursive schematic of orthorexia-related moral identity positioning

The present study examined how orthorexia-related discourse is produced and sustained in digital environments, with particular attention to the intersection of moralisation, identity formation, and platformed wellness culture. The findings suggest that many online narratives render orthorexia intelligible not simply as a set of dietary behaviours but as a morally saturated identity position, extending qualitative accounts that locate orthorexia within broader ideological formations of contemporary health culture (Fixsen et al., 2020). Figure 2 synthesises the findings as an inductively derived interpretive schematic, an organising device for the discursive configuration identified in the CDA subset rather than a causal model, diagnostic framework, or general theory of ON. The figure is not intended as a formal theoretical model or diagnostic framework, but as a heuristic representation of one prominent pattern observed across the corpus. Importantly, this configuration did not characterise all orthorexia-related discourse. Recovery-oriented narratives frequently disrupted moral binaries, questioned virtue-based framings of food, and explicitly worked to decouple self-worth from dietary purity. These counter-patterns indicate that moralised identity positioning is culturally available but not inevitable, and that the same discursive resources may be mobilised for competing ends.

The figure illustrates an inductively derived schematic representation of how moralisation of food, identity–diet linkage, digital reinforcement, and ego-syntonic distress can co-occur and mutually reinforce one another within online wellness discourse.

The figure illustrates how moralisation of food, ideological identity fusion, digital reinforcement, and ego-syntonic distress operate as a self-sustaining discursive system. Rather than forming a linear progression, these domains mutually reinforce one another, stabilising restrictive eating practices as morally justified, identity-defining, and resistant to pathologisation within digital wellness culture. Figure 2 is presented as an analytic and interpretive heuristic (i.e. a conceptual schematic), rather than as an empirical or causal model. It is not presented as evidence that ON “is” this system in general, but as a model of how a particular discursive configuration may operate online, alongside counter-configurations (e.g. recovery-oriented decoupling).

A core pattern across platforms was the routine framing of food in moral terms, with lexicons of purity, contamination, and virtue transforming dietary choices into ethical acts. This aligns with work arguing that orthorexia develops within cultural systems that treat health as moral righteousness and translate adherence to idealised rules into moral self-governance (Horovitz, 2025). In this corpus, moral and purity terms did not merely describe food; they organised self-evaluation and justified corrective restriction, a pattern consistent with research linking social media health content to orthorexic tendencies and to identity- and self-worth contingencies (Horovitz, 2025; Paul & Headley-Johnson, 2025; Salter & Dickson, 2021). Importantly, these observations are presented as discursive-construction patterns rather than as measures of symptom severity or evidence of causal platform effects.

The findings also resonate with broader medical-anthropological and sociological scholarship examining the relationship between food practices, moral selfhood, embodiment, and care. Some show how restrictive eating practices may become intertwined with valued forms of self-care and responsibility, rendering them difficult to recognise as harmful (Musolino et al., 2015). Similarly, others demonstrate how eating-disorder-related identities can be sustained through shared meanings and forms of belonging that complicate simple distinctions between pathology and agency (Eli & Lavis, 2022). These perspectives complement work positioning orthorexia within contemporary cultures of healthism, where food practices become vehicles for moral self-governance and identity formation (Hanganu-Bresch, 2020). The present findings extend this literature by illustrating how such processes are enacted, defended, and negotiated within digital wellness environments.

The data also showed that dietary ideology can be narratively fused with identity, positioning restrictive eating as ego-syntonic and resistant to critique. This resembles theoretical accounts in which lifestyle practices become central to belonging and self-definition, and it echoes qualitative findings that people engaged with orthorexia-related content often describe dietary discipline as integral to who they are (Gherasim et al., 2020; Horowitz et al., 2025; Sneijder & Molder, 2009). Related research on online health communities likewise suggests that shared language and norms can generate feedback loops that stabilise particular self-understandings (Xu et al., 2024). At the same time, the same discursive moves are also compatible with platform genre conventions and audience-facing performance demands, so the moral identity interpretation is treated as one plausible reading among others and is most strongly supported in the intensified CDA subset.

More broadly, scholarship beyond ON-specific research indicates that social media use can shape eating attitudes and well-being through normative beliefs and body-related cognition (Kovan & Yıldırım, 2025). Although this study did not model outcomes directly, the co-occurrence of moral framing and identity positioning suggests that platforms may amplify culturally resonant moral diets, thereby supporting rigid self-evaluative repertoires. This is consistent with concerns that weakly moderated online spaces can normalise extreme health practices (Lerman et al., 2024), while still falling short of permitting causal claims about exposure effects. The study therefore contributes to ongoing efforts to distinguish ON from other eating disorders by showing how “orthorexia” can be narratively organised around culturally sanctioned ideals of purity, discipline, and health knowledge rather than around appearance-driven motives (Fixsen et al., 2020), while also recognising possible overlap with other psychological processes such as perfectionism or obsessive–compulsive features (Cobzeanu et al., 2025). Because the material is publicly performed discourse, these distinctions are treated as differences in moral and narrative positioning rather than as clinical classification.

Crucially, the corpus was not discursively uniform. Recovery-oriented narratives more often rejected moral binaries, questioned virtue-signaling dynamics, or reframed health language as pragmatic rather than morally mandatory. These counter-patterns indicate that orthorexia discourse is heterogeneous and that the “moral identity” configuration identified here is best understood as a prominent, culturally available pathway rather than an invariant feature of orthorexia narratives.

From a clinical and preventive standpoint, the findings suggest that interventions may benefit from addressing moralised self-definitions and the discursive ecology that sustains them. These implications are discursively informed and hypothesis-generating, rather than prescriptive treatment recommendations. Standard cognitive-behavioural approaches may require augmentation with strategies that help decouple self-worth from dietary purity and reduce absolutist moral framing, while digital literacy approaches may support prevention by strengthening critical evaluation of online health rhetoric, consistent with work highlighting the double-edged psychosocial effects of social media nutrition content (Toğuç & Hökelek, 2025). These implications are offered as hypothesis-generating targets for practice rather than recommendations derived from outcome data.

The study’s limitations follow from its interpretive design and reliance on public texts: causal claims about social media effects are not possible, private or algorithmically curated experiences may be underrepresented, and discourse-based inference cannot adjudicate competing explanations such as anxiety management, influencer economics, or platform genre norms. Future work would benefit from designs that triangulate discourse with interviews or longitudinal/experimental approaches to test how shifts in exposure and language relate to changes in orthorexic cognition and behaviour, and to clarify when moralisation is central versus epiphenomenal. Additionally, the findings should not be interpreted as suggesting that all orthorexia-related experiences are organised through moral identity processes; rather, the analysis identifies one prominent and culturally available discursive configuration observed within this corpus.

### Contributions and Implications

The empirical case of orthorexia is thus used to sharpen and specify social-theoretical debates about healthism, moral regulation, and digitally mediated self-surveillance, rather than to adjudicate diagnostic status or estimate prevalence. Empirically, the manuscript’s added value lies in (a) platform-sensitive comparison across Reddit, YouTube, and blogs, and (b) explicit differentiation of genre orientations, lifestyle advocacy/education, ambivalent reflection, and recovery, showing that similar moral vocabularies do different interactional work depending on audience design and platform affordances. Conceptually, the contribution is not simply that ON discourse is moralised, but that moralisation can become identity-defensive and non-negotiable through patterned linkages among evaluation, self-positioning, and boundary work.

## Conclusion

This study demonstrates that orthorexia-related discourse is often constructed online as a morally saturated and identity-relevant position, in which food becomes an ethical object and dietary restraint is tied to virtue, integrity, and self-worth. By focusing on discursive mechanisms rather than diagnostic categories, the analysis clarifies how restrictive eating practices can remain ego-syntonic and resistant to change within digital wellness cultures. At the same time, the presence of counter-narratives highlights the heterogeneity of orthorexia discourse and cautions against treating moralisation as a defining feature of ON per se.

The findings suggest that orthorexia may function as a discursively organised identity possibility within some forms of digital wellness culture, helping explain both its cultural appeal and its resistance to pathologisation while pointing towards prevention and intervention strategies that address moralised self-concept alongside behavioural rigidity.

## Data Availability

The data analysed in this study consist of publicly available online texts (e.g. Reddit posts, YouTube transcripts, and publicly accessible blogs). Because the material involves potentially sensitive health-related narratives and to minimise the risk of re-identification, the compiled dataset is not publicly shared. Illustrative materials are presented in paraphrased or composite form within the article. Information about data sources and sampling procedures is provided in the Methods section.
